# Likelihood Ratio Testing under Measurement Errors

**DOI:** 10.3390/e20120966

**Published:** 2018-12-13

**Authors:** Michel Broniatowski, Jana Jurečková, Jan Kalina

**Affiliations:** 1 Faculté de Mathématiques, Laboratoire de Probabilité, Statistique et Modélisation, Université Pierre et Marie Curie (Sorbonne Université), 4 place Jussieu, 75252 Paris CEDEX 05, France; 2 Institute of Information Theory and Automation, The Czech Academy of Sciences, Pod Vodárenskou věží 4, 182 08 Prague 8, Czech Republic; 3 Faculty of Mathematics and Physics, Charles University, Sokolovská 83, 186 75 Prague 8, Czech Republic; 4 Institute of Computer Science, The Czech Academy of Sciences, Pod Vodárenskou věží 2, 182 07 Prague 8, Czech Republic

**Keywords:** measurement errors, robust testing, two-sample test, misspecified hypothesis and alternative, 2-alternating capacities

## Abstract

We consider the likelihood ratio test of a simple null hypothesis (with density $f_{0}$) against a simple alternative hypothesis (with density $g_{0}$) in the situation that observations $X_{i}$ are mismeasured due to the presence of measurement errors. Thus instead of $X_{i}$ for i=1,…,n, we observe $Z_{i}=X_{i}+\sqrt{\delta }V_{i}$ with unobservable parameter δ and unobservable random variable $V_{i}$. When we ignore the presence of measurement errors and perform the original test, the probability of type I error becomes different from the nominal value, but the test is still the most powerful among all tests on the modified level. Further, we derive the minimax test of some families of misspecified hypotheses and alternatives. The test exploits the concept of pseudo-capacities elaborated by Huber and Strassen (1973) and Buja (1986). A numerical experiment illustrates the principles and performance of the novel test.

## 1. Introduction

Measurement technologies are often affected by random errors; if the goal of the experiment is to compare two probability distributions using data, then the conclusion can be distorted if the data are affected by some measurement errors. If the data are mismeasured due to the presence of measurement errors, the statistical inference performed with them is biased and trends or associations in the data are deformed. This is common for a broad spectrum of applications e.g., in engineering, physics, biomedicine, molecular genetics, chemometrics, econometrics etc. Some observations can be even undetected, e.g., in measurements of magnetic or luminous flux in analytical chemistry when the flux intensity falls below some flux limit. Actually, we can hardly imagine real data free of measurement errors; the question is how severe the measurement errors are and what their influence on the data analysis is [1,2,3].

A variety of functional models have been proposed for handling measurement errors in statistical inference. Technicians, geologists, and other specialists are aware of this problem, and try to reduce the effect of measurement errors with various ad hoc procedures. However, this effect cannot be completely eliminated or substantially reduced unless we have some additional knowledge on the behavior of measurement errors.

There exists a rich literature on the statistical inference in the error-in-variables (EV) models as is evidenced by the monographs of Fuller [4], Carroll et al. [5], and Cheng and van Ness [6], and the references therein. The monographs [4] and [6] deal mostly with classical Gaussian set up while [5] discusses numerous inference procedure under semi-parametric set up. Nonparametric methods in EV models are considered in [7,8] and in references therein, and in [9], among others. The regression quantile theory in the area of EV models was started by He and Liang [10]. Arias [11] used an instrumental variable estimator for quantile regression, considering biases arising from unmeasured ability and measurement errors. The papers dealing with practical aspects of measurement error models include [12,13,14,15,16], among others. Recent developments in treating the effect of measurement errors on econometric models was presented in [17] or [18] The advantage of *rank and signed rank procedures* in the measurement errors models was discovered recently in [19,20,21,22,23,24]. The problem of interest in the present paper is to study how the measurement errors can affect the conclusion of the likelihood ratio test.

The distribution function of measurement errors is considered unknown, up to zero expectation and unit variance. When we use the the likelihood ratio test while ignoring the possible measurement errors, we can suffer a loss in both errors of the first and second kind. However, we show that under a small variance of measurement errors, the original likelihood ratio test is still most powerful, only on a slightly changed significance level.

On the other hand, we may consider the situation that $H_{0}$ or $H_{1}$ are classes of distributions of random variables $Z+\sqrt{\delta }V.$ Hence, both hypothesis and alternative are composite as families $H_{0}$ and $H_{1};$ if they are bounded by alternating Choquet capacities of order 2, then we can look for a minimax test based on the ratio of the capacities, and/over on the ratio of the pair of the least favorable distributions of $H_{0}$ and $H_{1}$, respectively (cf. Huber and Strassen [25]).

## 2. Likelihood Ratio Test under Measurement Errors

Our primary goal is to test the null hypothesis $H_{0}$ that independent observations $X={\left(X_{1},\ldots ,X_{n}\right)}^{⊤}$ come from a population with a density *f* against the alternative $H_{1}$ that the true density is g, where *f* and *g* are fixed densities of our interest. For the identifiability, we shall assume that *f* and *g* are continuous and symmetric around 0. Although the alternative is the main concern of the experimenter, some measurement errors or just the nature may cause the situation that the true alternative should be considered as composite. Specifically, $X_{1},\ldots ,X_{n},$ can be affected by additive measurement errors, what appears in numerous fields, as illustrated in Section 1.

Hence the alternative is $H_{1,\delta }$ under which the observations are $Z_{i,\delta }=X_{i}+\sqrt{\delta }V_{i},$ identically distributed with continuous density $g_{\delta }.$ Here, both under the hypothesis and under the alternative, $V_{i}$ are independent random variables, unobservable with unknown distribution, independent of $X_{i};\;i=1,\ldots ,n.$ The parameter δ>0 is also unknown, only we assume that $E\;V_{i}=0$ and $EV_{i}^{2}=1,$ for simplicity. The mismeasured, hence unobservable, $X_{i}$ are assumed to have the density *g* under the alternative. Quite analogously, the mismeasured observations lead to a composite hypothesis $H_{0,\delta }$ under which the density of observations $Z_{i,\delta }=X_{i}+\sqrt{\delta }V_{i}$ is $f_{\delta }$ while the $X_{i}$ are assumed to have density *f*.

If we knew $f_{\delta }$ and $g_{\delta },$ we would use the Neyman-Pearson critical region

(1) $$W=\left\{z:\sum_{i=1}^{n}\ln\left(\frac{g_{\delta }\left(z_{i}\right)}{f_{\delta }\left(z_{i}\right)}\right)\geq u\right\}$$

with *u* determined so that

$$P_{f_{\delta }}\left\{\sum_{i=1}^{n}\ln\left(\frac{g_{\delta }\left(z_{i}\right)}{f_{\delta }\left(z_{i}\right)}\right)\geq u\right\}=\alpha ,$$

with a significance level α. Evidently

$$\begin{array}{c} \int I\left[\sum_{i=1}^{n}\ln\left(\frac{g_{\delta }\left(z_{i}\right)}{f_{\delta }\left(z_{i}\right)}\right)\geq u\right]\prod_{i=1}^{n}g_{\delta }\left(z_{i}\right)dz_{i}=\int I\left[\sum_{i=1}^{n}\ln\left(\frac{g(x_{i})}{f(x_{i})}\right)\geq u\right]\prod_{i=1}^{n}g\left(x_{i}\right)dx_{i}\\ \int I\left[\sum_{i=1}^{n}\ln\left(\frac{g_{\delta }\left(z_{i}\right)}{f_{\delta }\left(z_{i}\right)}\right)\geq u\right]\prod_{i=1}^{n}f_{\delta }\left(z_{i}\right)dz_{i}=\int I\left[\sum_{i=1}^{n}\ln\left(\frac{g(x_{i})}{f(x_{i})}\right)\geq u\right]\prod_{i=1}^{n}f\left(x_{i}\right)dx_{i}. \end{array}$$

Indeed, notice that

$$\begin{array}{c} E_{g_{\delta }}\left\{I\left[\sum_{i=1}^{n}\ln\left(\frac{g_{\delta }\left(Z_{i}\right)}{f_{\delta }\left(Z_{i}\right)}\right)\geq u\right]|V_{1}=v_{1},\ldots ,V_{n}=v_{n}\right\}\\ =E_{g}\left\{I\left[\sum_{i=1}^{n}\ln\left(\frac{g(X_{i})}{f(X_{i})}\right)\geq u\right]|V_{1}=v_{1},\ldots ,V_{n}=v_{n}\right\}\;\forall v_{i}\in R,\;i=1,\ldots ,n, \end{array}$$

where the expectations are considered with respect to the conditional distribution; a similar equality holds for $f_{\delta }.$

Combining the integration transmission in the conditional distribution, we obtain

(2) $$\begin{array}{c} \int I\left[\sum_{i=1}^{n}\ln\left(\frac{g_{\delta }\left(x_{i}+\sqrt{\delta }V_{i}\right)}{f_{\delta }\left(x_{i}+\sqrt{\delta }V_{i}\right)}\right)\geq u\right]\prod_{i=1}^{n}f\left(x_{i}\right)dx_{i}\\ \neq \int I\left[\sum_{i=1}^{n}\ln\left(\frac{g(x_{i})}{f(x_{i})}\right)\geq u\right]\prod_{i=1}^{n}f\left(x_{i}\right)dx_{i}=\alpha , \end{array}$$

hence the size of the critical region *W* when used for testing $H_{0}$ against $H_{1}$ differs from α. Then we ask how the critical region *W* in (1) behaves when it is used as a test of $H_{0}.$ This problem we shall try to attack with an expansion of $f_{\delta },g_{\delta }$ in δ close to zero.

### Approximations of Densities

Put $f=f_{0},\;g=g_{0}$ the densities of *X* under the hypotheses and alternative, respectively. For the identifiability, we shall assume that $f_{0}$ and $g_{0}$ are continuous and symmetric around 0. Denote $f_{\delta }$ the density of $Z_{\delta }=X+\sqrt{\delta }V.$ This means that *X* is affected by an additive measurement error $\sqrt{\delta }\;V,$ where *V* is independent of *X* and $EV=0,\;EV^{2}=1,\;EV^{4}<\infty .$ Notice that if densities of *X* and *V* are strongly unimodal, then that of *Z* is also strongly unimodal (see [26]). Under some additional conditions on $f_{0},\;g_{0},$ we shall derive approximations of $f_{\delta }$ and $g_{\delta }$ for small δ>0. More precisely, we assume that both $f_{0}$ and $g_{0}$ have differentiable and integrable derivatives up to order 5. Then we have the following expansion of $f_{\delta }$ and a parallel result for $g_{\delta }$:

**Theorem** **1.** *Assume that $f_{0}$ and $g_{0}$ are symmetric around 0, strongly unimodal with differentiable and integrable derivatives, up to the order 5. Then, as δ↓0,*

(3) $$\begin{array}{c} f_{\delta }\left(z\right)=f_{0}\left(x+\sqrt{\delta }V\right)=f_{0}\left(x\right)+\frac{\delta }{2}\frac{d^{2}}{dz^{2}}f_{0}\left(x\right)+\frac{\delta^{2}}{4!}\frac{d^{4}}{dz^{4}}f_{0}\left(x\right)E\left(V^{4}\right)+o\left(\delta^{2}\right),\\ g_{\delta }\left(z\right)=g_{0}\left(x+\sqrt{\delta }V\right)=g_{0}\left(x\right)+\frac{\delta }{2}\frac{d^{2}}{dz^{2}}g_{0}\left(x\right)+\frac{\delta^{2}}{4!}\frac{d^{4}}{dz^{4}}g_{0}\left(x\right)E\left(V^{4}\right)+o\left(\delta^{2}\right) \end{array}$$

**Proof.**  Let $\varphi \left(u,\delta \right)=E\left\{e^{iuZ}\right\}$ be the characteristic function of Z. Then

$$\begin{array}{c} \varphi \left(u,\delta \right)=E\left\{e^{iuX}\right\}E\left\{e^{iu\sqrt{\delta }V}\right\}=\varphi \left(0,0\right)\varphi_{V}\left(u\sqrt{\delta }\right)\\ =\varphi \left(u,0\right)\left[1+\frac{1}{2}\delta {\left(iu\right)}^{2}+\frac{1}{4!}\delta^{2}{\left(iu\right)}^{4}E\left(V^{4}\right)+o\left(\delta^{2}\right)\right]\\ =\varphi \left(u,0\right)\left[1-\frac{\delta }{2}u^{2}+\frac{1}{4!}\delta^{2}u^{4}E\left(V^{4}\right)+o\left(\delta^{2}\right)\right], \end{array}$$

where $\varphi_{V}$ denotes the characteristic function of *V*. Taking the inverse Fourier transform on both sides, we obtain (3), taking the above assumptions on *V* into account. □

Consider the problem of testing the hypothesis $H_{0}$ that the observations are distributed according to density $f_{0}$ against the alternative $H_{1}$ that they are distributed according to density $g_{0}.$ Parallelly, we consider the hypothesis $H_{0,\delta }$ that observations are distributed according the $g_{\delta }$ against the alternative $H_{1,\delta }$ that the true density is $g_{\delta }.$ Let Φ(x) be the likelihood ratio test with critical region $W=\left\{x:\sum_{i=1}^{n}\ln\left(\frac{g_{0}\left(x_{i}\right)}{f_{0}\left(x_{i}\right)}\right)>u\right\}$ and the significance level α, and $\Phi^{*}=\Phi^{*}\left(z\right)$ be the test with critical region $W^{*}=\left\{z:\sum_{i=1}^{n}\ln\left(\frac{g_{0}\left(z_{i}\right))}{f_{0}\left(z_{i}\right)}\right)>u\right\}$ based on observations $z_{i}=x_{i}+\sqrt{\delta }V_{i},\;i=1,\ldots ,n.$ We know neither δ nor V, hence the test $\Phi^{*}$ is just an application of the critical region *W* for contaminated data $Z_{1},\ldots ,Z_{n}.$ Thus, due to our lack of information, we use the test Φ even for testing $H_{0,\delta }$ against $H_{1,\delta },$ and the performance of this test is of interest. This is described in the following theorem:

**Theorem** **2.** (Assume the conditions of Theorem 1). Then, as δ↓0, the test $\Phi^{*}$ is the most powerful even for testing $H_{0,\delta }$ against $H_{1,\delta },$ with a modified significance level satisfying

$$\alpha_{\delta }\leq \alpha +\frac{\delta }{2}\;|f_{0}^{\prime }\left(0\right)|+\frac{\delta^{2}}{24}\;EV^{4}\;\left|f_{0}^{\left(3\right)}\left(0\right)\right|+O\left(\delta \right).$$

**Proof.**  $$\begin{array}{c} E_{f_{0}}\Phi^{*}\left(X\right)=\int I\left[\ln\left(\frac{g_{0}\left(x+\sqrt{\delta }V\right)}{f_{0}\left(x+\sqrt{\delta }V\right)}\right)>u\right]f_{0}\left(x\right)dx\\ =\int I\left[\ln\left(\frac{g_{0}\left(x+\sqrt{\delta }V\right)}{f_{0}\left(x+\sqrt{\delta }V\right)}\right)>u\right]\frac{f_{0}\left(x\right)}{f_{0}\left(x+\sqrt{\delta }V\right)}f_{0}\left(x+\sqrt{\delta }V\right)dx\\ =\int I\left[\ln\left(\frac{g_{0}\left(x\right)}{f_{0}\left(x\right)}\right)>u\right]\frac{f_{0}\left(x-\sqrt{\delta }V\right)}{f_{0}\left(x\right)}f_{0}\left(x\right)dx. \end{array}$$

If $f_{0}$ is symmetric, then the derivative $f_{0}^{\left(k\right)}$ is symmetric for *k* even and skew-symmetric for *k* odd, k=1,…,4. Moreover, because $|f_{0}^{\prime }\left(x\right)|$ and $|f_{0}^{\left(3\right)}\left(x\right)|$ are integrable, then $\lim_{x\to \pm \infty }\left|f_{0}^{\prime }\left(x\right)\right|=0$ and $\lim_{x\to \pm \infty }\left|f_{0}^{\left(3\right)}\left(x\right)\right|=0.$ Hence, using the expansion (3), we obtain

$$\begin{array}{c} E_{f_{0}}\Phi^{*}\left(X\right)=E_{f_{0}}\Phi \left(X\right)+\int I\left[\ln\left(\frac{g_{0}\left(x\right)}{f_{0}\left(x\right)}\right)>u\right]\left(\frac{\delta }{2}f_{0}^{\prime \prime }\left(x\right)+\frac{\delta^{2}}{24}EV^{4}f^{\left(4\right)}\left(x\right)dx\right)+o\left(\delta^{2}\right)\\ \leq E_{f_{0}}\Phi \left(X\right)+\frac{\delta }{2}\;|f_{0}^{\prime }\left(0\right)|+\frac{\delta^{2}}{24}\;EV^{4}\;|f_{0}^{\left(3\right)}\left(0\right)|+o\left(\delta^{2}\right)=\alpha +O\left(\delta \right)\;as\;\delta ↓0. \end{array}$$

□

## 3. Robust Testing

If the observations are missmeasured or contaminated, we observe $Z_{\delta }=Z+\sqrt{\delta }V$ with unknown δ and unobservable *V* instead of *Z*. Hence, instead of simple $f_{0}$ and $g_{0},$ we are led to composite hypothesis and alternative H and K. Following [25], we can try to find suitable 2-alternating capacities, dominating H and K and to construct a pertaining minimax test. As before, we assume that *Z* and *V* are independent, $EV=0,\;EV^{2}=1$, and $EV^{4}<\infty .$ Moreover, we assume that $f_{0}$ and $g_{0}$ are symmetric, strongly unimodal and differentiable up to order 5, with derivatives integrable and increasing distribution functions $F_{0}$ and $G_{0},$ respectively. The measurement errors *V* are assumed to satisfy

(4) $$1\leq EV^{4}\leq K$$

with a fixed K,0<K<∞. Hence the distribution of *V* is restricted to have the tails lighter than *t*-distribution with 4 degrees of freedom. We shall construct a pair of 2-alternating capacities around specific subfamilies of $f_{0}$ and $g_{0}.$

Let us determine the capacity around $g_{0}$; that for $f_{0}$ is analogous. By Theorem 1 we have

$$g_{\delta }\left(z\right)=g_{0}\left(z\right)+\frac{\delta }{2}\frac{d^{2}}{dz^{2}}g_{0}\left(z\right)+\frac{\delta^{2}}{4!}\frac{d^{4}}{dz^{4}}g_{0}\left(z\right)E\left(V^{4}\right)+o\left(\delta^{2}\right),\;as\;\delta ↓0.$$

We shall concentrate on the following family $K^{*}$ of densities (similarly for $f_{0}$):

(5) $$\;K^{*}=\left\{g_{\delta ,\kappa }^{*}:\;g_{\delta ,\kappa }^{*}\left(z\right)=g_{0}\left(z\right)+\frac{\delta }{2}g_{0}^{\prime \prime }\left(z\right)+\kappa \frac{\delta^{2}}{24}g_{0}^{\left(4\right)}\left(z\right)\;|\;\delta \leq \Delta ,1\leq \kappa \leq K\right\}$$

with fixed suitable Δ,K>0.

Indeed, under our assumptions, each $g_{\delta ,\kappa }^{*}\in K^{*}$ is a positive and symmetric density satisfying

$$\underset{\delta \leq \Delta ,\kappa \leq K}{\sup}\underset{z\in \{R}{\sup}\left|g_{\delta ,\kappa }^{*}\left(z\right)-g_{0}\left(z\right)\right|\leq CK\Delta^{2}+o\left(\Delta^{2}\right)$$

for some C,0<C<∞.

Let $G_{\delta ,\kappa }^{*}\left(B\right),\;B\in B,$ be the probability distribution induced by density $g_{\delta ,\kappa }^{*}\in K^{*},$ with B being the Borel σ-algebra. Then the set function

(6) $$w\left(B\right)=\left\{\begin{array}{ccc} \sup\left\{G^{*}\left(B\right):G^{*}\in K^{*}\right\} & \mathrm{if} & B\neq \emptyset \\ 0 & \mathrm{if} & B=\emptyset \end{array}\right.$$

is a pseudo-capacity in the sense of Buja [27], i.e., satisfying

**(a)**  w(∅)=0,w(Ω)=1
**(b)**  w(A)≤w(B)∀A⊂B
**(c)**  $w\left(A_{n}\right)↑w\left(A\right)\;\forall A_{n}↑A$
**(d)**  $w\left(A_{n}\right)↓w\left(A\right)\;\forall A_{n}↓A\neq \emptyset$
**(e)**  w(A∪B)+w(A∩B)≤w(A)+w(B).

Analogously, consider a density $f_{0},$ symmetric around 0 and satisfying the assumptions of Theorem 1 as a simple hypothesis. Construct the family $H^{*}$ of densities and the corresponding family of distributions $\left\{F_{\delta ,\kappa }^{*}\left(\cdot \right),\;\delta \leq \Delta ,\;\kappa \leq K\right\}$ similarly as above. Then the set function

(7) $$v\left(B\right)=\left\{\begin{array}{ccc} \sup\left\{F^{*}\left(B\right):F^{*}\in H^{*}\right\} & \mathrm{if} & B\neq \emptyset \\ 0 & \mathrm{if} & B=\emptyset \end{array}\right.$$

is a pseudo-capacity in the sense of Buja [27].

Buja [27] showed that on any Polish space exists a (possibly different) topology which generates the same Borel algebra and on which every pseudo-capacity is a 2-alternating capacity in the sense of [25].

Let us now consider the problem of testing the hypothesis $H=\left\{F^{*}\in H^{*}|F^{*}\left(\cdot \right)\leq v\left(\cdot \right)\right\}$ against the alternative $K=\left\{G^{*}\in K^{*}|G^{*}\left(\cdot \right)\leq w\left(\cdot \right)\right\},$ based on an independent random sample $Z_{1},\ldots ,Z_{n}.$ Assume that $H^{*}$ and $K^{*}$ satisfy (5). Then, following [27] and [25], we have the main theorem providing the minimax test of H against K with significance level α∈(0,1):

**Theorem** **3.** *The test*

$$\phi \left(z_{1},\ldots ,z_{n}\right)=\begin{array}{ccc} 1 & \mathrm{if} & \prod_{i=1}^{n}\pi \left(z_{i}\right)>C\\ \gamma & \mathrm{if} & \prod_{i=1}^{n}\pi \left(z_{i}\right)=C\\ 0 & \mathrm{if} & \prod_{i=1}^{n}\pi \left(z_{i}\right)<C \end{array}$$

*where π(·) is a version of $\frac{dw}{dv}\left(\cdot \right)$ and C and γ are chosen so that $E_{v}\phi \left(Z\right)=\alpha ,$ is a minimax test of H against K of level α.*

## 4. Numerical Illustration

We assume to observe independent observations $Z_{1,\delta },\cdots ,Z_{n,\delta }$ for i=1,⋯,n, where $Z_{i,\delta }=X_{i}+\sqrt{\delta }V_{i}$ as described in Section 3, where $X_{1},\cdots ,X_{n}$ are independent identically distributed (with a distribution function *F*) but unobserved. Let us further denote by Φ the distribution function of N(0,1) and by $\Phi_{\sigma }^{*}$ the distribution function of $N(0,\sigma^{2})$. The primary task here is to test $H_{0}:\;F\equiv \Phi$ against

$$H_{1}:\;F\left(x\right)=\left(1-\lambda \right)\Phi \left(x\right)+\lambda \Phi_{\sigma }^{*}\left(x\right),\;x\in R,$$

with a fixed σ>1 and λ∈(0,1). We perform all the computations using the R software [28].

To describe our approach to computing the test, we will need the notation for the set of pseudo-distribution functions corresponding to the set of pseudo-densities $H^{*}$ denotes as

$${\tilde{H}}^{*}=\left\{F_{\delta ,\kappa }^{*}:\;F_{\delta ,\kappa }^{*}\left(z\right)=\Phi \left(z\right)+\frac{\delta }{2}f_{0}^{\prime }\left(z\right)+\kappa \frac{\delta^{2}}{24}f_{0}^{\left(3\right)}\left(z\right)\;|\;\delta \leq \Delta ,\;1\leq \kappa \leq K\right\},$$

where Φ denotes the distribution function of N(0,1) distribution. Under the alternative, the set analogous to $K^{*}$ is defined as

$${\tilde{K}}^{*}=\left\{G_{\delta ,\kappa }^{*}:\;G_{\delta ,\kappa }^{*}\left(z\right)=G_{0}\left(z\right)+\frac{\delta }{2}g_{0}^{\prime }\left(z\right)+\kappa \frac{\delta^{2}}{24}g_{0}^{\left(3\right)}\left(z\right)\;|\;0\leq \delta \leq \Delta ,\;1\leq \kappa \leq K\right\}.$$

Our task is to approximate

(8) $$v\left((-\infty ,z)\right)=\sup\left\{F_{\delta ,\kappa }^{*}\left(z\right);F_{\delta ,\kappa }^{*}\in {\tilde{H}}^{*}\right\},\;z\in R,$$

and

(9) $$w\left((-\infty ,z)\right)=\sup\left\{G_{\delta ,\kappa }^{*}\left(z\right);G_{\delta ,\kappa }^{*}\in {\tilde{K}}^{*}\right\},\;z\in R.$$

Here, the functions $F_{\delta ,\kappa }^{*}\left(z\right)$ and $G_{\delta ,\kappa }^{*}\left(z\right)$ are evaluated over a grid with step 0.05. Then, the maximization in (8) and (9) is performed for values of *z* over the grid and over four boundary values of ${\left(\delta ,\kappa \right)}^{T}$, which are equal to ${\left(0,0\right)}^{T}$, ${\left(0,K\right)}^{T}$, ${\left(\Delta ,0\right)}^{T}$, and ${\left(\Delta ,K\right)}^{T}$. Additional computations with 10 randomly selected pairs of ${\left(\delta ,\kappa \right)}^{T}$ over δ∈[0,Δ] and κ∈[0,K] revealed that the optimum is attained in one of the boundary values. Further, the Radon-Nikodym derivatives of *V* and *W* are estimated by a finite difference approximation in order to compute the test statistic.

The test rejects $H_{0}$ if the test statistics $\prod_{i=1}^{n}\pi \left(z_{i}\right)$ exceeds a critical value, which (as well as the *p*-value) can be approximated by a Monte Carlo simulation, i.e., by a repeated random generating random variables $X_{1},\cdots ,X_{n}$ under $H_{0}$, and we generate them 10,000 times here.

We perform the following particular numerical study. We compute the critical value of the α-test for n=20 (or n=40), λ=0.25, $\sigma^{2}=3$, Δ=0.2, K=1.1, and α=0.05. Further, we are interested in evaluating the probability of rejecting this test for data generated from

(10) $$F\left(x\right)=\left(1-\tilde{\lambda }\right)\Phi \left(x\right)+\tilde{\lambda }\Phi_{\tilde{\sigma }}^{*}\left(x\right),\;x\in R,$$

with different values of $\tilde{\lambda }$ and ${\tilde{\sigma }}^{2}$. Its values are shown in Table 1 (for n=20) and Table 2 (for n=40), which are approximated using (again) 10,000 randomly generated variables from (10). The boldface numbers are equal to the power of the test (under the simple $H_{1}$). The proposed test seems meaningful, while its power is increased for n=40 compared to n=20; in addition, the power increases with an increasing $\tilde{\lambda }$ if ${\tilde{\sigma }}^{2}$ is retained; and the power also increases with an increasing ${\tilde{\sigma }}^{2}$ if $\tilde{\lambda }$ is retained.

## 5. Conclusions

The likelihood ratio test of $f_{0}$ against $g_{0}$ is considered in the situation that observations $X_{i}$ are mismeasured due to the presence of measurement errors. Thus instead of $X_{i}$ for i=1,…,n, we observe $Z_{i}=X_{i}+\sqrt{\delta }V_{i}$ with unobservable parameter δ and unobservable random variable $V_{i}$. When we ignore the presence of measurement errors and perform the original test, the probability of type I error becomes different from the nominal value, but the test is still the most powerful among all tests on the modified level.

Under some assumptions on $f_{0}$ and $g_{0}$ and for $\delta <\Delta ,\;EV^{4}\leq K,$ we further construct a minimax likelihood ratio test of some families of distributions of the $Z_{i}=X_{i}+\sqrt{\delta }V_{i},$ based on the capacities of the Huber-Strassen type. The test treats the composite null and alternative hypotheses, which cover all possible measurement errors satisfying the assumptions. The advantage of the novel test is that it keeps the probability of type I error below the desired value (α=0.05) across all possible measurement errors. The test is performed in a straightforward way, while the user must specify particular (not excessively large) values of Δ and *K*. We do not consider this a limiting requirement, because parameters corresponding to the severity of measurement errors are commonly chosen in a similar way in numerous measurement error models [5,23] or robust optimization procedures [29]. The critical value of the test can be approximated by a simulation. The numerical experiment in Section 4 illustrates the principles and performance of the novel test.

## Figures and Tables

**Table 1:** Probability of rejecting the test in the simulation with n=20.

Value of $\tilde{\lambda }$	Value of ${\tilde{\sigma }}^{2}$			
	3	4	5	6
0.25	0.39	0.52	0.61	0.67
0.35	0.50	0.67	0.75	0.81
0.45	0.61	0.76	0.85	0.89

**Table 2:** Probability of rejecting the test in the simulation with n=40.

Value of $\tilde{\lambda }$	Value of ${\tilde{\sigma }}^{2}$			
	3	4	5	6
0.25	0.55	0.73	0.82	0.87
0.35	0.72	0.86	0.93	0.96
0.45	0.82	0.94	0.97	0.99
